# A Comparative Evaluation for Biologic Width following Surgical Crown Lengthening Using Gingivectomy and Ostectomy Procedure

**DOI:** 10.1155/2012/479241

**Published:** 2012-08-26

**Authors:** Kiran Kumar Ganji, Veena Ashok Patil, Jiji John

**Affiliations:** ^1^Department of Periodontics, College of Dental Sciences & Hospital, Rau. Devi Ahilya University, Indore, Madhya Pradesh, India; ^2^ Department of Periodontics, H.K.E. Society's S. Nijlingappa Institute of Dental Sciences and Research Centre, Gulbarga. Rajiv Gandhi University of Health Sciences, Bangalore, Karnataka, India

## Abstract

Surgical crown lengthening has been proposed as a means of facilitating restorative procedures and preventing injuries in teeth with structurally inadequate clinical crown or exposing tooth structure in the presence of deep, subgingival pathologies which may hamper the access for proper restorative measures. Histological studies utilizing animal models have shown that postoperative crestal resorption allowed reestablishment of the biologic width. However, very little has been done in humans. *Aims*. The purpose of the study was to evaluate the potential changes in the periodontal tissues, particularly the biologic width, following surgical crown lengthening by two surgical procedures before and after crown placement. *Methods and Material*. Twenty (20) patients who needed surgical crown lengthening to gain retention necessary for prosthetic treatment and/or to access caries, tooth fracture, or previous prosthetic margins entered the study. The following parameters were obtained from line angles of treated teeth (teeth requiring surgical crown lengthening) and adjacent sites: Plaque and Gingival Indices (PI) & (GI), Position of Gingival Margin from reference Stent (PGMRS), Probing depth (PD), and Biologic Width (BW). *Statistical Analysis Used*. Student “*t*” Test. *Results*. Initial baseline values of biologic width were 2.55 mm (Gingivectomy procedure B1 Group) and 1.95 mm (Ostectomy procedure B2 Group) and after surgical procedure the values were 1.15 mm and 1.25 mm. *Conclusions*. Within the limitations of the study the biologic width, at treated sites, was re-established to its original vertical dimension by 3 months. Ostectomy with apically positioned flap can be considered as a more effective procedure than Gingivectomy for Surgical Crown Lengthening.

## 1. Introduction

The preservation of a healthy periodontium is critical for the long-term success of a restored tooth. Dentists must constantly balance the restorative and esthetic needs of their patients with periodontal health [1]. One factor that is of particular importance is the potential damage that results in the periodontium when margins are placed subgingivally.

Garguilo et al. [2] described the dimensions and relations of the dentogingival junction in humans; the biologic width is the zone of the root surface coronal to the alveolar crest to which the junctional epithelium and connective tissue are attached; it averages 2.04 mm. These dimensions may vary from tooth to tooth, but it is present in all healthy dentition [3]. It was shown that crown margins positioned subgingivally were associated with the most gingival inflammation leading to violation of biologic width, whereas supragingivally located crown margins were associated with the least gingival inflammation. Supragingival placement of restoration margins allows for ease of impression making, cleansing [4], and detection of secondary caries and is associated with maintainable probing depths [4, 5]. Subgingival restorations can have damaging effects on the neighboring hard and soft tissues, especially when they encroach on the junctional epithelium and supracrestal connective tissue [6]. These subgingivally placed restorations have been associated with gingival inflammation, loss of connective tissue attachment, and bone resorption [3, 7, 8].

Allen [9] reports that wherever the biologic width is violated, there is a reaction by the periodontium. Alveolar bone will resorb inconsistently in an attempt to provide space for a new connective tissue attachment, which will result in an increase in probing depth.

It would therefore seem to be more prudent to increase the dimension of the clinical crown through surgical crown lengthening rather than risk a violation of the periodontium (biologic width) by injudicious subgingival tooth preparations [10, 11]. It also provides clinical tooth structure to enable the placement of margins either coronal or equigingival. To avoid these potential problems to the supporting structures of teeth, surgical crown lengthening can provide adequate clinical crown structure.

Surgical crown lengthening has been proposed as a means of facilitating restorative procedures and preventing injuries in the teeth with structurally inadequate clinical crown or exposing tooth structure in the presence of deep subgingival pathologies that may hamper the access for proper restorative measures. Surgical crown lengthening can be performed by gingivectomy and ostectomy with apically positioning flap, in order to facilitate restorative procedures and to prevent periodontal injuries in teeth with structurally inadequate clinical crowns; the apically positioned flap technique with osseous resection has been recommended [11–13].

Osseous resective procedures used in periodontal therapy have been shown to be efficient in stabilizing periodontal destruction [14] when osseous resective procedures are used in combination with an apically positioned flap for pocket reduction or elimination, much of the pocket regrowth has been attributed as stated by Ochsenbein [15] to the “dynamic behavior” of the gingival that prefers to live at or near the CEJ and that always seems determined to return to its original preoperative architectural form.

Smukler and Chaibi described how a predetermined entity called supracrestal gingival tissue differs from site to site, will reform after surgical excision and how the “regrowth” will be dictated by the underlying anatomy of the dental and osseous units [16]. In the past literature, however, sufficient information has not been provided regarding the dimension of the postsurgical soft tissue modifications or the amount of time necessary to achieve the complete healing of the periodontal tissues; therefore, the stability of the soft tissue levels exists.

The few clinical studies on periodontal tissue alterations, which occur during healing after surgical crown lengthening, reported conflicting results [17, 18]. Vander Velden [17] observed 3 years after surgery a considerable amount of coronal regrowth of the interproximal gingival tissue from the level where the osseous crest was located after surgery [17]. On the contrary, Brägger et al. found over a 6-month healing period after surgical crown lengthening stable periodontal tissues, with minimum changes in the gingival margin levels from surgery to the end of the study [18].

Previously reported clinical studies [16–18] on surgical crown lengthening have followed positional changes of the free gingival margin immediately after surgery and during healing but have not focused on the biologic width. A few histological studies utilizing animal models have shown postoperative crestal resorption after denudation [19] and scaling and root planing [20] allowed the reestablishment of connective tissue attachment. However, very little work has been done to conform these results in human clinical trials.

Therefore, the purpose of the study was to evaluate the positional changes of the periodontal tissues, particularly the biologic width, for a period of 6 months where in two surgical crown lengthening procedures gingivectomy and ostectomy with apically positioned flap were performed and assessment of changes in periodontal tissues was done prior to and after crown placement.

## 2. Subjects and Methods

The clinical study included 30 patients, of 20 to 40 years of age (mean age 30), selected on the basis of various conditions hampering proper restorative measures for placement of full crown, one or more teeth and requiring surgical crown lengthening to

- gain retention in sites with insufficient supracrestal tooth structure necessary for prosthetic reconstruction,
- gain accessibility to deep, subgingivally located lesions or preexisting faulty preparation margins for restorative treatment.

The patients were selected from the Department of Prosthodontics and Periodontics, H.K.E.S's S.N. Dental College Gulbarga. The study and the procedures were explained to the patients and a written informed consent was taken.

### 2.1. Inclusion Criteria

1. Systemically and periodontally healthy patients.
2. Endodontically treated or grossly destructed tooth or part of fixed partial denture requiring full crown.
3. Short clinical crown length but adequate root length.

### 2.2. Exclusion Criteria

1. Systemically and/or periodontally compromised patients.
2. Nonrestorable dentition.
3. Unfavourable crown-root ratio.
4. Orthodontic intrusions.

The selected patients are divided into 2 groups randomly.

1. Group A (control).
2. Group B (experimental).
  - Group B1.
  - Group B2.

**Group A**

In this group about 10 patients were selected who actually required crown lengthening but the crown lengthening was not done and crown margins were placed subgingivally.

**Group B1**

In this group about 10 patients were selected requiring crown lengthening and surgical procedure was carried out only by soft tissue removal, that is, gingivectomy and after surgical procedure margins of the restoration were placed supragingivally.

**Group B2**

In this group about 10 patients were selected requiring crown lengthening and surgical procedure was carried out by soft and hard tissue removal, that is, ostectomy with apically positioned flap and after surgical procedure margins of the restoration were placed supragingivally.

After an initial examination and treatment planning session, each patient received detailed instruction in proper self-performed plaque control measures and underwent full-mouth scaling/root planing and removal of marginal irritants. After 1 week of plaque control supervision, the patients were recalled for a baseline examination. At the baseline examination, the following parameters were recorded for each tooth at 4 sites (mesiobuccal, distobuccal, buccal, and lingual) of both the groups.

1. Schick Ash Plaque Index (PI) [3, 5].
2. Loe and Silness Gingival Index (GI).
3. Position of the gingival margin (PGM), determined by assessing the distance from a fixed reference point using acrylic stent.
4. Probing depth.
5. Clinical attachment level.
6. Biologic width (calculated by subtracting the measurement of bone sounding from the gingival margin).

In order to standardize probe placement and angulation during measurements, a full acrylic stent was fabricated for the required tooth and vertical grooves were created at appropriate interproximal sites. All measurements were obtained with a standardized Williams Graduated probe and rounded up to the nearest millimeter.

### 2.3. Position of Gingival Margin

It is estimated using the acrylic stent fabricated preoperatively. The vertical grooves made on the acrylic stent guide the probe to maintain the site specificity. The stent is marked with the horizontal line, which acts as a reference mark. The position of the gingival margin is calculated by measuring the distance between the reference mark on the stent placed on the tooth and the gingival margin at various interval of examination.

### 2.4. Determination of Biologic Width

 It is determined using Williams Graduated Periodontal Probe. Firstly the depth of the gingival sulcus is measured and anesthesia is given then bone sounding is done, that is, the distance from the gingival margin to the alveolar bone is measured. Then, the biologic width is determined by subtracting the gingival sulcus depth from the distance from the gingival margin to the alveolar bone.

Following the baseline examination sequential surgical therapy was carried out as shown in Table 1.

After treatment plan presentation, patients were provided information about the study and indicated willingness to participate, by providing written informed consent. An alginate impression was taken of each arch to be surgically treated in order to fabricate customized probing stents. Probing stents were made from self-cure acrylic resin material using dough method. Stents were trimmed to the height of contour of all teeth, and grooves were placed at the sites to be measured with a 1169-fissure bur. To improve visualization, the apical margin of the probing stent was traced with a black permanent marker. Using the probing stent, the following baseline measurements were taken for each tooth at the surgical appointment prior to administering local anesthetic:

1. the probing depth,
2. relative attachment level from base of sulcus to stent,
3. the distance from stent to gingival margin.

All measurements of the parameters were taken by single examiner using a Williams Graduated periodontal probe.

Surgical procedure was accomplished under local anesthesia. The following guidelines were taken into consideration.

1. The first guideline was to place the alveolar crest at a level of at least 3 mm from the anticipated crown margin.
2. To allow sufficient room for tooth preparation the second guideline required was to leave wherever possible at least 9 mm of clinical crown height coronal to the osseous crest. This calculation was derived from anticipating that a tooth in occlusion and awaiting a crown restoration would require 2 mm of occlusal reduction for restorative space, 4 mm of axial wall length, and 3 mm distance from restorative margin to the bone.
3. The third surgical guideline was to place the flap margin 3 mm apical to the anticipated restorative margin following suturing.

## 3. Statistical Analysis

Statistical analysis is done by using Student's  *t*-test unpaired because of independent variables. Test of significance between B1 and B2 Groups is calculated. The average of all weeks and further average of all averages are calculated and are designated as X1^−^ and X2^−^. Standard error is calculated and designated as SE.

Comparison between the weeks of all the parameters is done by Student's paired  *t*-test because of same samples that are dependent.

## 4. Results

30 patients completed this study and no complications related to the surgery or prosthetic treatment were observed.

### 4.1. Control Group A

#### 4.1.1. Probing Depth (Figure 1)

**Figure 1:**
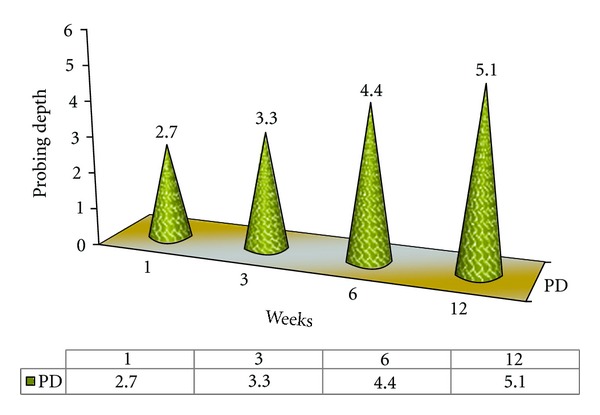
Mean probing depth (PD) values of Control Group A during various intervals of weeks.

The peak hike in probing depth values might be a result of loss of attachment, which is due to violation of biologic width. In other words margins placed subgingivally are associated with increased probing depth as shown in Figure 1 associated with violation of biologic width. The periodontium tried to maintain the same biologic width and shifted apically at the expense of crestal bone loss.

### 4.2. Results of Experimental Group B1 (Figure 2)

**Figure 2:**
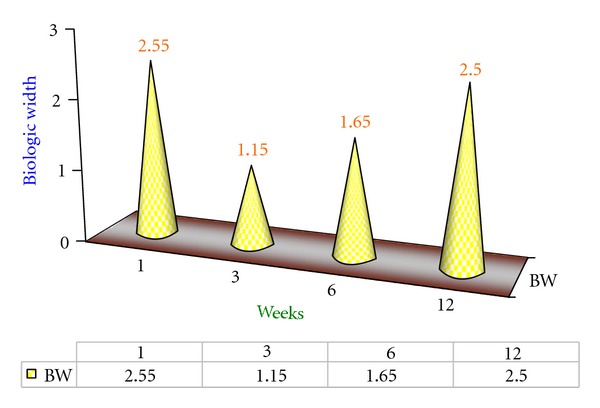
Mean biologic width values of B1 Group at various intervals of weeks.

Biologic width at the 12th week was 2.5 mm reestablishing the baseline value as shown in Figure 2.

#### 4.2.1. Position of Gingival Margin from Reference Stent (PGMRS) (Figure 3)

The change in mean values from the 3rd week to the 6th week indicates coronal movement of the gingival margin.

### 4.3. Results of Experimental Group B2 (Figure 4)

It is demonstrated that once the biologic width is established after 6 weeks postsurgically the value remained same and there was no significant difference between the 6th and 12th week as shown in Figure 4.

### 4.4. Position of Gingival Margin from Reference Stent of Group B2 (Figure 5)

There was significant difference of mean values of PGMRS between the 1st and 3rd week. At the 6th week the mean value was about 9.1, which then increased to 9.4 by the 12th week. The mean values of PGMRS at the 3rd and 12th weeks were nearly the same, that is, 9.3 and 9.4; hence no significant differences in PGRMS were found between the 3rd, 6th, and 12th weeks. The result indicates that there was no coronal movement of gingival margin from reference stent during an evaluation period of about 3 months and stability of the free gingival margin was noticed as shown in Figure 5.

### 4.5. Comparison of Mean Biologic Width Values of B1 and B2 Groups (Figure 6)

During the 6th to 12th weeks there was significant difference in mean biologic width of B1 Group, that is, 1.65 mm to 2.5 mm but there was no significant difference in B2 Group as shown in Figure 6.

### 4.6. Comparison of Mean Values of PGMRS of B1 and B2 Groups (Figure 7)

There was significant change in B1 Group between the 3rd week and the 6th week when compared with B2 group. Then, there was no significant change in PGMRS during the 6th and 12th weeks in both the groups as shown in Figure 7.

### 4.7. Probing Depth Values (Figure 8)

Value of  *T*  table (*t*  tab) is 2.262. “*t*” table = 2.262 at 5% level of significance. The tabulated values in the chart are “*t*” calculated values (*t*  cal). If  *t*  cal >  *t*  tab then there is significant change. Note that if  *t*  cal <  *t*  tab, then there is no significant change.

The  *T*  cal value for probing depth for the 1st and 3rd weeks is 3.6742, for the 1st and 6th weeks is 11.130, and for the 1st and 12th weeks is 1.0854. Since all the values are greater than  *T*  tab value, there is a significant difference in probing depth values at baseline and the 12th week examination. This indicates progressive loss of attachment over a period of time as shown in Figure 8.

### 4.8. Interweek Comparison of Biologic Width for B1 Group: (Figure 9)

 The  *t*  calculated value for biologic width between the 1st and 3rd week is 8.5732 and for the 1st and 6th week is 4.3223; hence there is significant difference in comparison of Biologic values between the 1st, 3rd, and 6th week (because the  *t*  calculated >  *t*  table since  *t*  tab is 2.262). In comparison of the 1st to 12th week there is no significant change in biologic width values since  *t*  calculated is less than the  *t*  table value (*t*  cal 0.1901 <  *t*  Table 2.262). Hence it can be concluded that once the biologic width was established it's dimension of remained constant during an evaluation period of up to 12 weeks after gingivectomy procedureas shown in Figure 9.

Value of “*t*” table (*t*  tab) is 2.262. “*t*” table = 2.262 at 5% level of significance. The above-tabulated values are “*t*” calculated values (“*t*” cal). If  *t*  cal >  *t*  tab then there is significant change. Note that if  *t*  cal <  *t*  tab, then there is no significant change.

### 4.9. Interweek Comparison of Biologic Width for B2 Group: (Figure 10)

The “*t*” calculated value for biologic width between the 1st and 3rd week is 3.8221 and for the 1st and 6th weeks is 0.8018. Hence there is significant difference in comparison of biologic values between the 1st and 3rd week (because the “*t*” calculated > “*t*” table since “*t*” tab is 2.262) and there is no significant difference in comparison of biologic width between the 3rd and 6th week (because the “*t*” calculated < “*t*” table since  *T*  tab is 2.262). In comparison of the 1st to 12th week there is significant change in biologic width values since “*t*” calculated is greater than the “*t*” table value (*t*  cal 2.400 >  *t*  Table 2.262).

### 4.10. Interweek Comparison of PGMRS for B2 Group: (Figure 10)

The “*t*” cal values for PGMRS during the 1st and 3rd week is 19.240, during the 1st and 6th week is 28.169, and the 1st to 12th week is 27.299. Hence there is significant change from the 1st to 12th week of evaluation since  *t*  cal. >  *t*  tab. Hence it can be concluded that the PGMRS remained stable after the ostectomy with apically positioned flap procedure was performed as shown in Figure 10.

Value of “*t*” table (*t*  tab) is 2.262. “*t*” table = 2.262 at 5% level of significance. The tabulated values are “*t*” calculated values (*t*  cal) If  *t*  cal >  *t*  tab then there is significant change Note that if  *t*  cal <  *t*  tab, then there is no significant change.

### 4.11. Tissue Rebound following Surgery (Figure 11)

**Figure 3:**
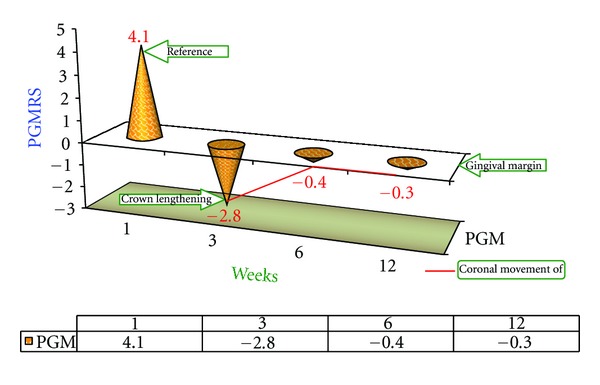
Deviation graph of position of gingival margin from reference stent (PGMRS) (B1 Group) at various intervals of weeks.

**Figure 4:**
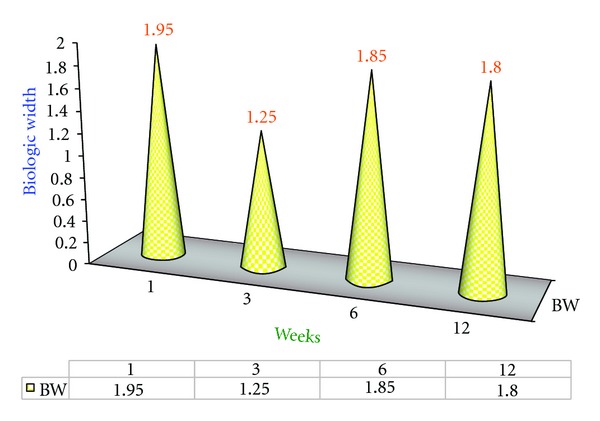
Mean biologic width (BW) values of B2 Group at various intervals of weeks.

**Figure 5:**
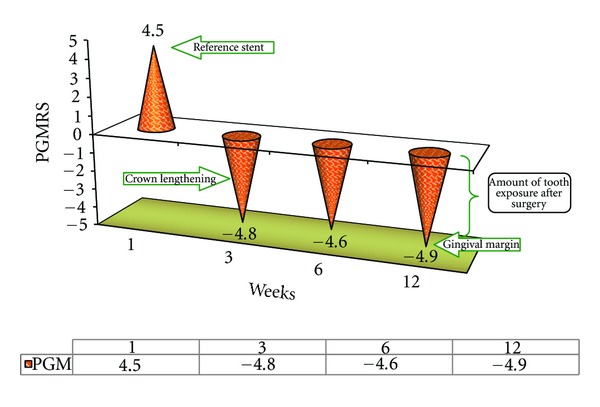
Deviation graph of position of gingival margin from reference stent (PGMRS) (B2 Group) at various intervals of weeks.

**Figure 6:**
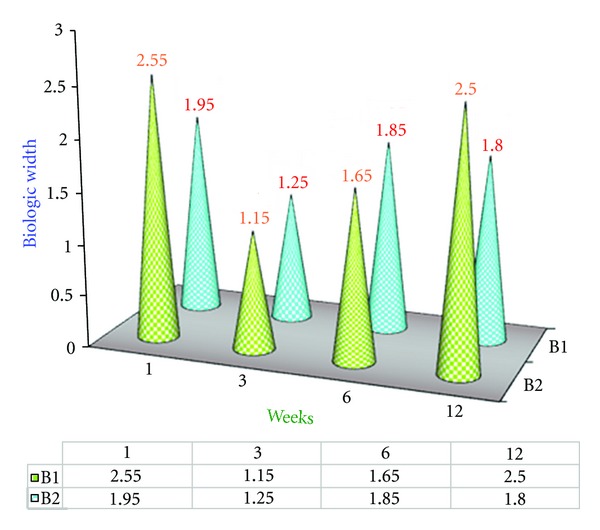
Comparison of mean biologic width values of B1 and B2 Groups.

**Figure 7:**
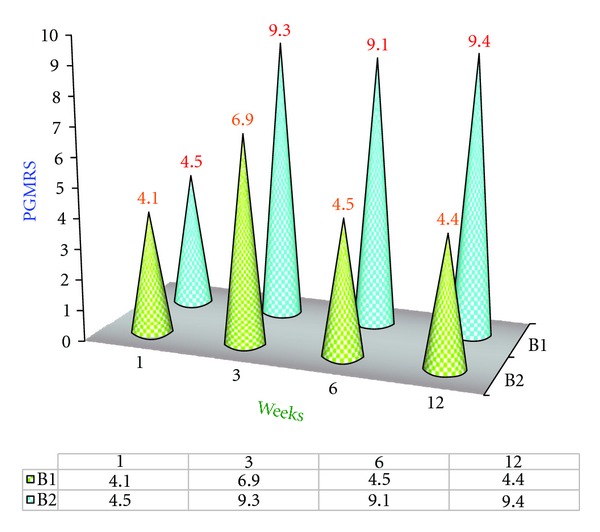
Comparison of mean position of gingival margin from reference stent (PGMRS) of B1 and B2 Groups.

**Figure 8:**
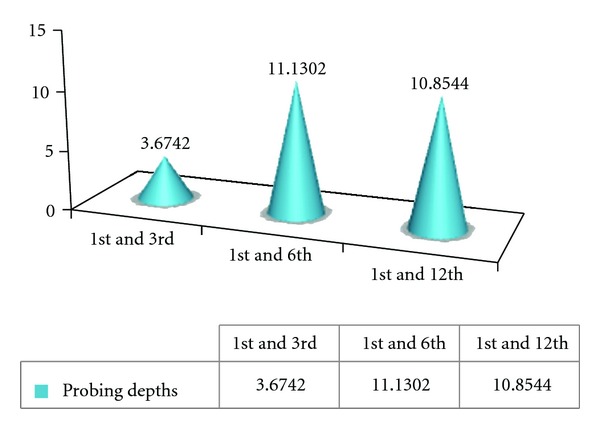
Graphical representation of tabulated probing depth values of Control Group A.

**Figure 9:**
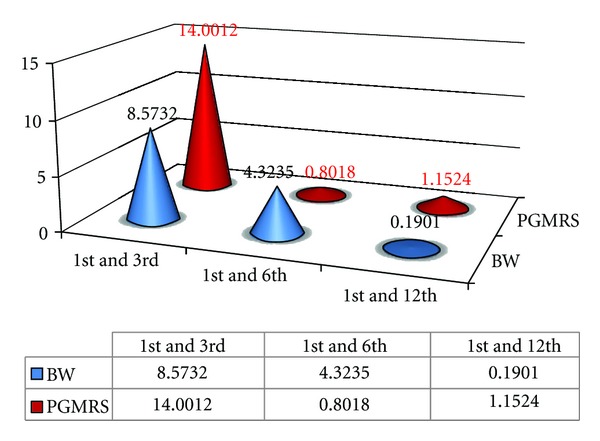
Comparison of tabulated values of biologic width (BW) and position of gingival margin from reference stent (PGMRS) with that of interweek intervals of Group B1.

**Figure 10:**
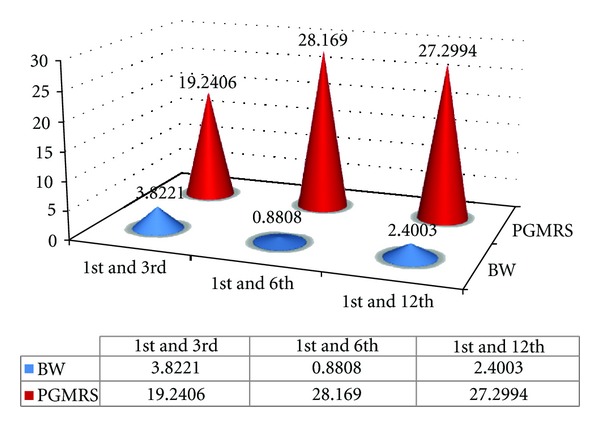
Comparison of Tabulated values of Biologic Width (BW) and Position of GIngival Margin from Reference Stent (PGMRS) with that of Interweek intervals of Group B2.

In order to investigate the phenomenon of tissue rebound following crown lengthening surgery (gingivectomy Versus ostectomy with apically positioned flap) the changes in PGMRS from baseline to the 12th week postoperatively have been plotted in Figures 3 and 5 and the “*t*” calculated values are tabulated.

**Figure 11:**
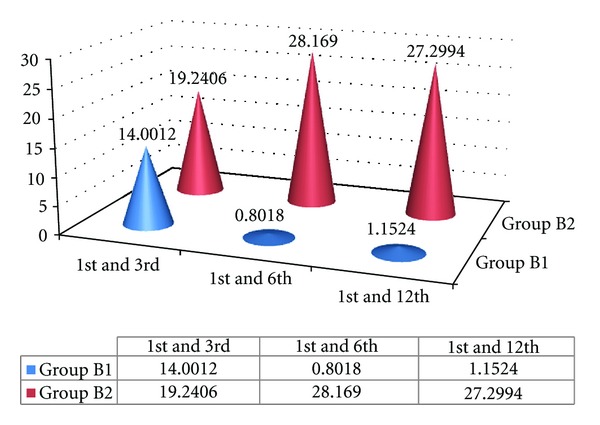
Comparison of Tabulated values of Position of GIngival Margin from Reference Stent (PGMRS) within B1 and B2 Group at various intervals of weeks.

After reviewing the number and distribution of data points, it can be concluded that amount of tissue rebound by the 12th week was the greatest in case of gingivectomy procedure with no significant changes in  *T*  cal value (1.1524) between the 1st and 12th week but the amount of tissue rebound was minimal in case of ostectomy with apically positioned flap with significant difference of  *T*  cal value (27.299) between the 1st 12th week. This relationship held true regardless of treatment group or whether the sites were interproximal or facial/lingual as shown in Figure 11.

Hence it can be concluded that the ostectomy with apically positioned flap procedure is more superior to gingivectomy procedure for surgical crown lengthening.

## 5. Discussion

Over the years, dentists have had difficulty in correctly relating the restorative margin placement to the periodontal apparatus; in the prosthetic treatment of advanced periodontal-prosthetic case, many failures have been due to the incorrect prosthetic management of the periodontal soft and hard tissues. These failures frequently have been blamed on poor oral hygiene and poor cooperation by the patient but this is not always true. It is because of violation of biologic width due to subgingival margin placement.

At the majority of sites in this study, the biologic width after surgical crown lengthening was significantly smaller compared to baseline. These changes in biologic width ranged between 1.25 to 1.8 mm in teeth treated with ostectomy procedure and 1.15 to 1.65 in case of teeth treated with gingivectomy procedure examined postsurgically. These findings are consistent with previous studies. Lanning et al. [1] demonstrated that after surgical crown lengthening, the biologic width at treated sites was reestablished to its original vertical dimension by 6 months. In addition a consistent 3 mm gain of coronal tooth structure was observed at the 3rd and 6th week examination.

A notable trend in this study was that the biologic width at all sites from the 3rd week to 12th week increased (i.e., approached baseline measurements). This is attributed to a slight gain in attachment level and apical displacement of the bone level. Oakley et al. [20] and Carnevale et al. [19] reported that bone resorption following surgical crown lengthening provides supracrestal tooth structure for the attachment of connective tissue, leading to reestablishment of the biologic width. The values of biologic width at the 3rd week were significantly different compared to baseline. In other words, the original dimension of biologic width was reestablished at treated sites after 12 weeks irrespective of the procedure used for surgical crown lengthening (either gingivectomy or ostectomy with Apically positioned flap).

The probing depths at all sites after surgery were not significantly different from baseline; this is consistent with other reports on surgical crown lengthening [21, 22].

The literature is inconsistent as to the advantages of manual versus controlled-force probes in terms of improving intraexaminer reproducibility [23–25]. In this study a manual probe offered greater practicality in obtaining measurements, particularly bone level via transgingival probing to determine biologic width, and therefore was the chosen method of obtaining the selected clinical parameters over time. In addition, measurements were obtained by only one examiner with a standardized periodontal probe utilizing reference stents.

In this study, the position of the free gingival margin, attachment and bone levels remained stable from 3rd to 12th week in case of teeth treated for surgical crown lengthening using ostectomy with apically positioned flap procedure. These findings are consistent with previous studies [18]. However, other contradicting studies have found positional changes of the periodontal tissues, during this time period [19, 22, 26].

The results of the present clinical investigation demonstrated that during 3-month healing period following surgical crown lengthening with apically positioned flap and osseous resection the marginal periodontal tissues showed no distinct tendency to grow in a coronal direction. But in case of gingivectomy the marginal periodontal tissues showed distinct tendency to grow in a coronal direction. In other words, the amount of the available crown length that increased from the presurgical level was 4.9 mm in case of ostectomy procedure and 0.3 mm in case of gingivectomy procedure. The postsurgical soft tissue remodeling occurred in conjunction with positive clinical measurements as shown by low Plaque and Gingival index scores throughout the study. It was also observed that the probing depth values tended to return to the presurgical values, with no difference between the baseline (interproximal 2.7 mm/buccal lingual 1.4 mm) and final examination (interproximal 2.8 mm, buccal lingual 1.3 mm). However, a difference was found between the clinical attachment level measurements obtained at the completion of the study and those recorded presurgically revealing an expected loss of clinical attachment in case of ostectomy procedure. These findings may suggest a tendency of the periodontium to reform a new “physiological” supracrestal gingival unit. The regrowth of the soft tissue from the level where the osseous crest was defined at surgery had already begun 1 month after surgery when the gingival margin reached about 60% of its final coronal position at interproximal sites and about 40% at buccal/lingual sites in case of gingivectomy procedure. These findings were in accordance with the study done by Lindhe and Nyman [27]. They proposed that during active periodontal treatment the position of gingival margin was shifted in an apical direction, but this displacement was to some extent compensated by a coronal regrowth during postoperative maintenance care period.

The factors influencing the amount of coronal displacement of the marginal periodontal tissues seemed to be related to the different tissue biotypes. Patients with thick tissue biotype demonstrated significantly more coronal soft tissue regrowth than patients with thin biotype due to the natural biological differences in inter-individual patterns of healing responses. Many factors seem to contribute to the maintenance of tooth structure gained through surgical crown lengthening procedures. Individual patient healing characteristics, reformation of biologic width, adequacy of positive osseous architecture created during surgery, timing of restorative procedures, and postoperative plaque control may be among these factors. Another factor may be the position of the flap margin after surgery, which was examined in the present study.

The reason for these opposite patterns of marginal periodontal tissue alterations after surgical crown lengthening may be due to the differences in the interpretation and/or execution of the surgical technique, which is assumed to be an apically positioned flap with osseous resection. Within the limits of the present study it can be concluded that for surgical crown lengthening ostectomy with apically positioned flap is much more a superior procedure than gingivectomy.

Alterations of the periodontal tissues similar to these found in the present report and the Vander Velden study [17] were observed by different authors [28–30] following treatment of intrabony defects by the apically positioned flap technique with osseous recontouring. In these studies, the authors found that gingival margin after apically positioned flap procedures and osseous recontouring shifted during 6 to 12 months of healing to a more coronal position [28–30] and that, after this period, it remained unchanged during 5 to 7 years of maintenance [29, 30], demonstrating a predictable stability in properly maintained patients. Hence within the limitations of this study, it can be concluded that the ostectomy with apically positioned flap procedure is superior to gingivectomy procedure for surgical crown lengthening.

The most important question is how long a clinician should wait after surgical crown lengthening procedure to begin restorative procedures to ensure stable results?

Infact this question is still a controversial issue as many authors quote range of 1 month or 3 months or up to 6 months. More and more clinical research is still needed to come to conclusion on this issue.

## Figures and Tables

**Table 1:** 

Timing of surgical procedure, crown placement, and clinical parameters assessment	Group A	Group B1	Group B2
After oral prophylaxis	Clinical parameters were assessed	(1) Clinical parameters were assessed	(1) Clinical parameters were assessed
		(2) Gingivectomy was carried out	(2) Ostectomy with Apically positioned flap was carried out

3 weeks after surgical procedure and before crown placement	Clinical parameters were assessed and tooth preparation followed by final prosthesis was made	Clinical parameters were assessed and tooth preparation followed by final prosthesis was made	Clinical parameters were assessed and tooth preparation followed by final prosthesis was made

6 weeks after crown placement	Clinical parameters were assessed	Clinical parameters were assessed	Clinical parameters were assessed

12 weeks after crown placement	Clinical parameters were assessed	Clinical parameters were assessed	Clinical parameters were assessed
